# Visual analysis on the research of monocarboxylate transporters based on CiteSpace

**DOI:** 10.1097/MD.0000000000027466

**Published:** 2021-11-05

**Authors:** Feifei Li, Shuqi Wang, Youlong Yao, Xueming Sun, Xiaoyan Wang, Ning Wang, Yulin You, Yanli Zhang

**Affiliations:** a School of Pharmaceutical Sciences, Shandong University, Jinan, China; b Department of computer science, Jinan Vocational College, Shandong, China; c Weifang Yidu Central Hospital, Weifang, Shandong, China; d Qilu Hospital of Shandong University, Jinan, China.

**Keywords:** CiteSpace, monocarboxylate transports, visual analysis

## Abstract

**Background:**

Monocarboxylate transports (MCTs), a family of solute carrier protein, play an important role in maintenance of cellular stability in tumor cells by mediating lactate exchange across membranes. The objective of this paper is to evaluate the knowledge structure, development trend, and research hotspot of MCTs research field systematically and comprehensively.

**Methods:**

Based on the 1526 publications from 2010 to 2020 retrieved from “Web of Science Core Collection” (WoSCC), we visually analyzed the MCTs research in terms of subject category, scientific collaboration network, keywords, and high-frequency literature using CiteSpace.

**Results:**

The number of publications exhibits an upward trend from 2010 to 2020 and the top 5 countries in the MCTs research were the United States, China, Japan, Germany, and England. Visser TJ was the most prolific author, while Halestrap AP was the most influential author with the highest citations. Analysis of the 7 cluster units from the co-cited references and keywords revealed that high expression of MCTs induced by oxidative stress and glycolysis was the pivotal point in the MCTs research field, while regulation of metabolism in tumor microenvironment, prognostic markers of cancer, and targeted inhibitors are the top 3 research frontiers topics.

**Conclusion:**

This study will help the new researcher to understand the MCTs related field, master the research frontier, and obtain valuable scientific information, thus providing directions for follow-up research.

## 1 Introduction

Abnormal intracellular energy metabolism, such as a dramatically higher glucose consumption rate, is one of the major characteristics of tumor cells.^[1,2]^ The phenomenon that tumor cells prefer to rely on aerobic glycolysis to generate the energy needed for cellular processes is known as “Warburg effect.” It is characterized by high glucose uptake, active glycolysis, and high production of lactate even in the presence of sufficient oxygen.^[3,4]^ In order to maintain intracellular enhanced glycolysis and physiological pH, the tumor cells need to excrete lactate into the extracellular microenvironment to prevent the occurrence of intracellular acidosis and cell death.^[5,6]^

Monocarboxylate transporters (MCTs) are proton-linked membrane carriers and participate in multiple vital metabolic pathways because of their involvement in transport of monocarboxylates, such as lactate, pyruvate, as well as ketone bodies, across membranes.^[7]^ (aka SLC16A1) Isoform 1 in the MCT superfamily (MCT1) and (aka SLC16A3) Isoform 4 in the MCT superfamily (MCT4) are 2 major lactate transporters under both physiological and pathological conditions.^[8–10]^ MCT1 is expressed in almost all tissues and its major physiological role is to facilitate lactate entry into or efflux out of cells depending on their metabolic state. However, MCT4 is distributed mainly in various glycolytic tissues such as white skeletal muscle fibers, astrocyte, white blood cells, and chondrocytes.^[11–14]^ Lactate shuttle in tumor cells, which is mainly mediated by MCT1 and MCT4, is important for their survival. Efflux of intracellular lactate to the extracellular environment leads to a decrease in its pH value, making the tumor cells more aggressive by stimulating its potential of metastasis.^[15–17]^ Therefore, the MCTs are potential targets in anti-cancer therapy, making its research of great significance.

CiteSpace, also named “Atlas of Scientific Knowledge,” is a visualized analysis software developed in the context of bibliometric analysis and data visualization, exhibiting structure, law, and distribution of the research area in a visualization way.^[18,19]^ Currently, visual analysis of the MCTs research is still limited. In the present study, we aimed to employ CiteSpace to analyze the retrieved literature in MCTs research field in a visualization way, in order to describe a collaboration network and detect the research hotspots and frontiers.

## 2 Methods

### 2.1 Data source

The literature in this paper were retrieved from Web of Science, and the search criterion were set up as follows: Web of Science Core Collection (WoSCC): citation index (SCI-EXPANDED, SSCI, A&HCI, CPCI-S, CPCI-SSH, and ESCI); language was “English” and document type was “All document types”; the time span was set as 2010 to 2020; TS = (“monocarboxylate transporter” or “monocarboxylic acid transporter” or “SLC16” or “SLC16 transporter” or “lactate transporter” or “lactate shuttle” or “monocarboxylate transporters” or MCTs). Under the above conditions, 3115 bibliography were retrieved on June 28, 2020. All records in our search results were exported in plain text format. After removing duplicates, content discrepancies, and other records, 1526 valid records with the document type as the article were imported into the CiteSpace for visual analysis finally.

### 2.2 Analysis method

Raw data were verified manually. Duplicates, other document type (including comments, conference summaries, books, etc) and irrelevant literature were deleted. Effective literatures were then visualized and analyzed using CiteSpace 5.7.R1. The parameters of CiteSpace were established as follows: the time slices value was set as 1 by default, which means that we specify 1 year as the length of a single time slice. One node type was selected at a time, including country, institution, author, cited author, cited journal, category, keyword, and cited reference in this study. The value of Top N was set as 50, which means that the 50 top-cited articles were selected for each time slice. The ethical approval was not applied in current study because there was no patient's privacy or clinical samples.

## 3 Results

### 3.1 Distribution of scientific output

**Figure 1:**
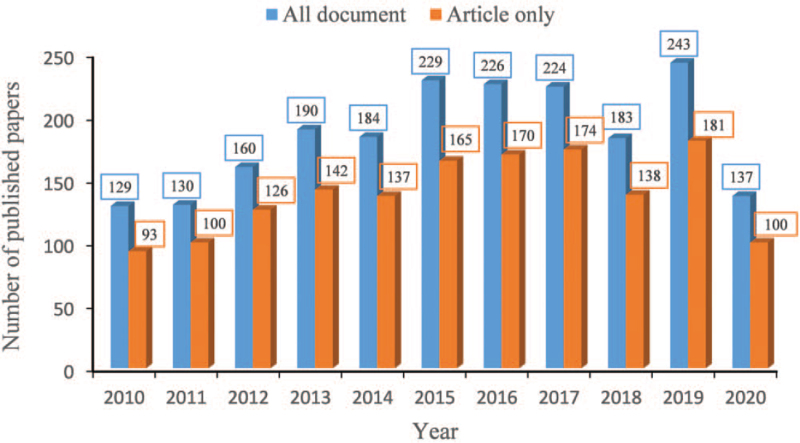
The annual number of publications on MCTs research. The abscissa in the figure represents the year, and the ordinate represents the number of published papers. MCTs = monocarboxylate transports.

The scientific output in MCTs research field by year is presented in Fig. 1. The number of publications from 2010 to 2015 exhibits an upward trend. In the following 3 years, it declines slightly, but increases to a new height in 2019 (243). An increasing trend for MCTs research is expected in the future.

### 3.2 Distribution of category

**Figure 2:**
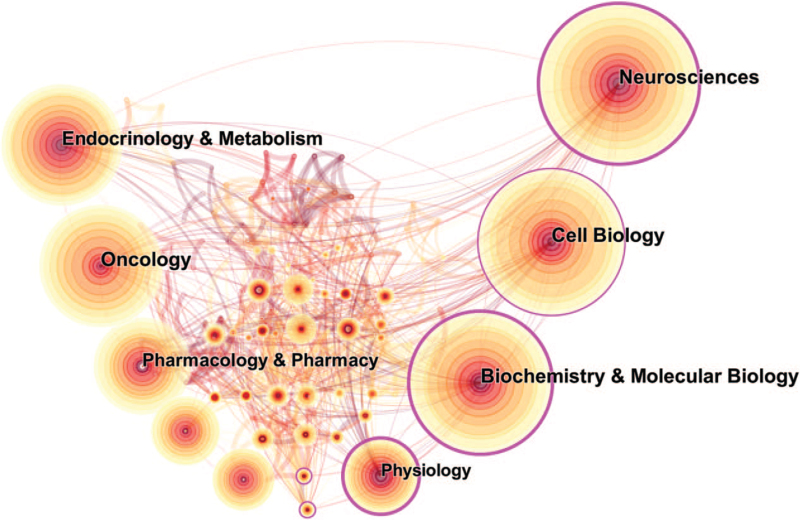
Visualization of the research field. (The size of a circle is proportional to the amount of literature of the category. The colors of rings correspond to the year. The purple rims of circles represent the high betweenness centralities. The thickness of the line is proportional to the relevance of different research areas.).

According to CiteSpace analysis, the 1526 articles related to MCTs research mainly focused on 3 aspects: expression of MCTs in tumor cells, the regulation of cell metabolism by MCTs, and the use of MCTs as a therapeutic target. As shown in Fig. 2, Neurosciences (240), Cell Biology (224), Biochemistry and Molecular biology (219), Oncology (192), and Endocrinology & Metabolism (184) are the top 5 research directions in the MCTs field (Supplemental_Dataset_S1 is CiteSpace data about distribution of category).

### 3.3 Distribution of countries and institutions

**Figure 3:**
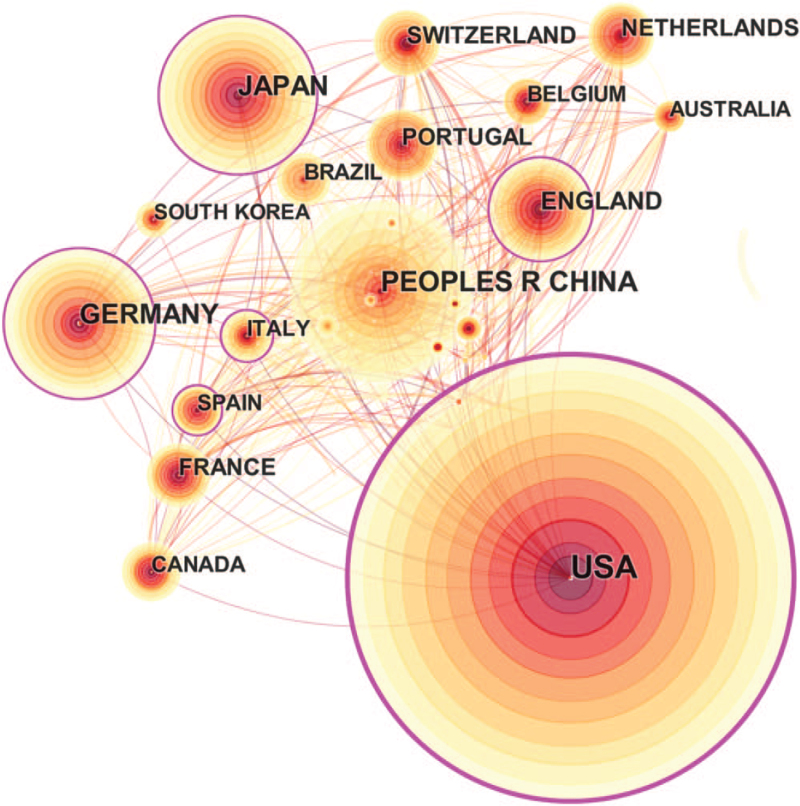
The network of contributing countries.

The quantity of publications in 1 country is an important criterion indicating its research strength in the specific field. The country collaboration network of MCTs research constructed by CiteSpace consists of 63 nodes and 420 links. As shown in Fig. 3, the size of a circle reflects the number of publications in each country or region. Betweenness centrality is an indicator that reflects the significance of a node in the network, and >0.1 can be called key nodes.^[20]^ USA ranked first with 453 publications that accounts for 29.8% of the total publishing papers, followed by China (197) and Japan (170) (Table 1). USA has collaborated with most of the countries in the past decade and has worked closely with China in recent years. In addition to the developed countries that provide the power of scientific research and innovation, the volume of publications in China, as a developing country, has increased dramatically in the last 4 years, indicating increasing concern on MCTs related research areas (Supplemental_Dataset_S2 is CiteSpace data about distribution of countries).

**Table 1:** The top 10 countries, institutions, and authors contributed to publications on MCTs research from 2010 to 2020.

Country	Count	Institution	Count	Author	Count
USA	453	Univ Minho (Portugal)	47	Visser TJ (Netherlands)	44
China	197	Univ Lausanne (Sweden)	39	Baltazar F (Portugal)	38
Japan	170	Univ Porto (Portugal)	34	Morris ME (USA)	27
Germany	167	Hokkaido University (Japan)	34	Pellerin L (Sweden)	26
England	112	ICVS 3Bs PT Govt Associate Lab (Portugal)	33	Pinheiro C (Brazil)	24
Netherlands	81	Univ Copenhagen (Denmark)	29	Visser WE (Netherlands)	19
Switzerland	80	Thomas Jefferson University (USA)	25	Becker HM (Germany)	16
Portugal	80	Erasmus MC (Netherlands)	24	Peeters RP (Netherlands)	16
France	74	Erasmus Univ (Netherlands)	23	Schweizer U (Germany)	15
Canada	70	Barretos Canc Hosp (Brazil)	23	Kobayashi M (Japan)	15

In the aspect of contribution of institutions, University of Minho in Portugal stands out with the largest circle of 47 publications, followed by University of Lausanne (39) and University of Porto (34) (Table 1). Among the top-10 institutions, 3 institutions were in Portugal and their mutual collaboration is close, indicating that the Portugal research institutions are more active than those other countries in MCTs related research because of their excellent research background. However, in general, most of the collaboration remains domestic. International collaboration still needs to be strengthened in the future (Supplemental_Dataset_S3 is CiteSpace data about distribution of institutions).

### 3.4 Distribution of authors, cited authors, and journals

**Figure 4:**
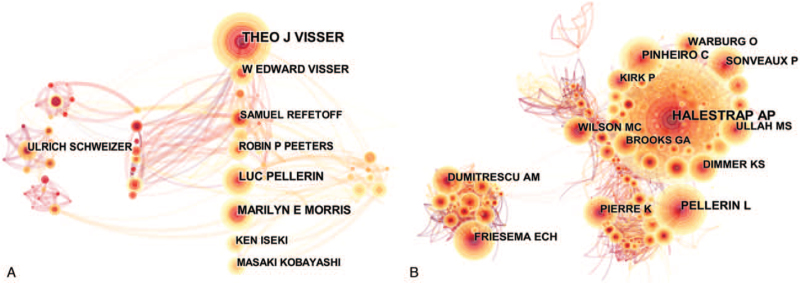
A. Co-authorship network on MCTs. The size of the nodes represents the number of publications published by the authors. B. Co-citation map of authors on MCTs. The size of the nodes represents the frequency of co-citation authors. MCTs = monocarboxylate transports.

The network of author contribution and collaboration was depicted in Fig. 4A. Four hundred twenty four nodes and 830 links were observed with 424 authors included. The graph shows that there are obvious groupings among the authors, mainly because the cooperation is based on geographical and institutional attributes, and the thicker the lines are, the higher the co-occurrence intensity is. The most productive author was Visser Theo J (44) from the Department of Internal Medicine, Erasmus University Medical Center, who focused the functions of (aka SLC16A2) Isoform 4 in the MCT superfamily (MCT8) and its role in abnormal thyroid hormone metabolism.^[21,22]^ Then the author followed by was Baltazar Fatima (38), who was based at Life and Health Sciences Research Institute (ICVS), at University of Minho, and performed research on abnormal expression of MCT as target in cancer drug therapy.^[23–25]^ Morris Marilyn E (27), from the State University of New York, investigated the transport mechanism of γ-hydroxybutyric acid (GHB) in mammalian cells.^[26,27]^ Pellerin Luc (26), from the University of Lausanne, examined the role of MCTs in the central nervous system.^[28,29]^ Pinheiro Celine (24), also based at ICVS, focused on the MCTs expression in human tumors and its prognostic value.^[30]^ (shown in Table 1) (Supplemental_Dataset_S4 is CiteSpace data about distribution of authors and cited authors).

Author co-citation refers to the fact that 2 articles of 2 different authors are cited by 1 article at the same time. These articles are also used as indicators of scientific competence and relevance. In the graph, the size of the nodes represents the citation frequency, which is used to evaluate the strength of an author's influence and authority. Meanwhile, nodes with high betweenness centrality indicate that the author plays a key role in the research field and makes significant contributions to its development. The network of influential authors is shown in Fig. 4B. Among the top 10 authors in terms of citation frequency, Halestrap AP, Ullah MS, and Wilson MC all come from England (Table 2), suggesting that England makes a significant contribution in the field of MCTs research in the past decade. Halestrap AP, from University of Bristol, is the most cited author on the research of mechanism underlying metabolites transport via MCTs as well as its biochemical characters and distribution in different tissues, and thus builds a theoretical foundation for following researchers.^[14,31,32]^

**Table 2:** Information on the most influential authors and top 10 journals with most published documents.

Cited authors	Citations	Institution	Journal	Frequency	IF (2019)	JCR region
Halestrap AP	557	University of Bristol	Journal of Biological Chemistry	989	4.238	Q2
Pellerin L	247	Univ Lausanne	Proc Natl Acad Sci USA	779	9.412	Q1
Pinheiro C	210	Barratos Cancer Hospital	Biochemical Journal	644	4.097	Q2
Friesema ECH	190	Erasmus MC	PLoS One	639	2.74	Q2
Sonveaux P	178	University of Leuven	Science	190	41.845	Q1
Ullah MS	162	University of Bristo	Journal of Clinical Investigation	553	11.864	Q1
Pierre K	156	Univ Lausanne	Nature	511	42.778	Q1
Dumitrescu AM	146	University of Chicago	Journal of Physiology-London	510	4.547	Q1
Dimmer KS	145	Univ Tubingen	Cancer Research	505	9.727	Q1
Wilson MC	145	University of Bristol	Cell	482	38.637	Q1

The co-citation of journals reflects the relevance of various types of journal and discipline. The distribution of knowledge base in a research field can be obtained by co-citation analysis. A co-citation network of journals was depicted by CiteSpace and 609 nodes and 5488 links were observed. Journals with top 10 citation frequency among the 609 journals included were listed in Table 2. Journal of Biological Chemistry (989 co-citations) was in the first place. In addition, articles published in journal with a high impact factor (>30), such as Cell, Nature, Sciences and Cancer Research, also have high citation frequency and create a certain amount of influence. All these journals constitute the research network in this field, which reflects the research foundation of MCTs family (Supplemental_Dataset_S5 is CiteSpace data about distribution of co-cited journals).

### 3.5 Analysis of co-occurring keywords and burst terms

**Figure 5:**
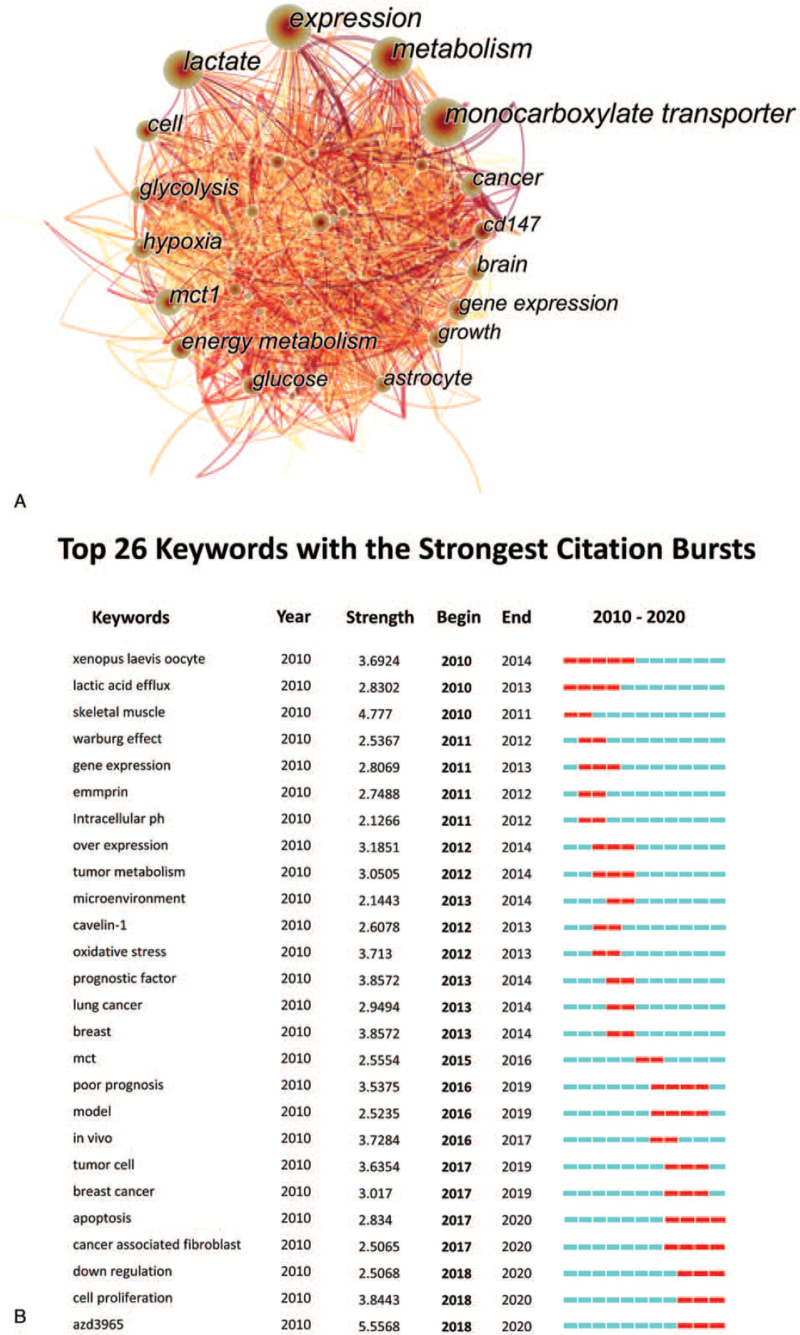
A. The network of keywords. B. Top 26 keywords with the strongest citation bursts on the research of MCTs during 2010 to 2020. MCTs = monocarboxylate transports.

The network of keywords is a highly generalized and concentrated description of research topics, reflecting the current research hotspots in the field. Four hundred seventy three nodes and 3910 links compose the network of keyword distribution, in which the size of the nodes is proportional to frequency and thickness of links represents the collaboration degree of 2 nodes (Fig. 5A). “Expression” ranked top with a frequency of 403, followed by “monocarboxylate transporter” (348) and “metabolism” (316) (Supplemental_Dataset_S6 is CiteSpace data about distribution of keywords).

Burst keywords are regarded as indicators of dramatic changes in the number of the citations in a certain period and can be used to detect the rise and decline of a keyword. Burst of keywords have 2 key aspects: the strength and duration of the burst, with the former representing the intensity of the burst, and the latter including the beginning and the end of the burst time, as shown by the red line in Fig. 5B. Twenty-six burst keywords were identified, with “xenopus laevis oocyte,” “lactic acid,” “skeletal muscle,” “warurg effect,” and “gene expression” ranking top 5.

### 3.6 Analysis of co-cited references

**Figure 6:**
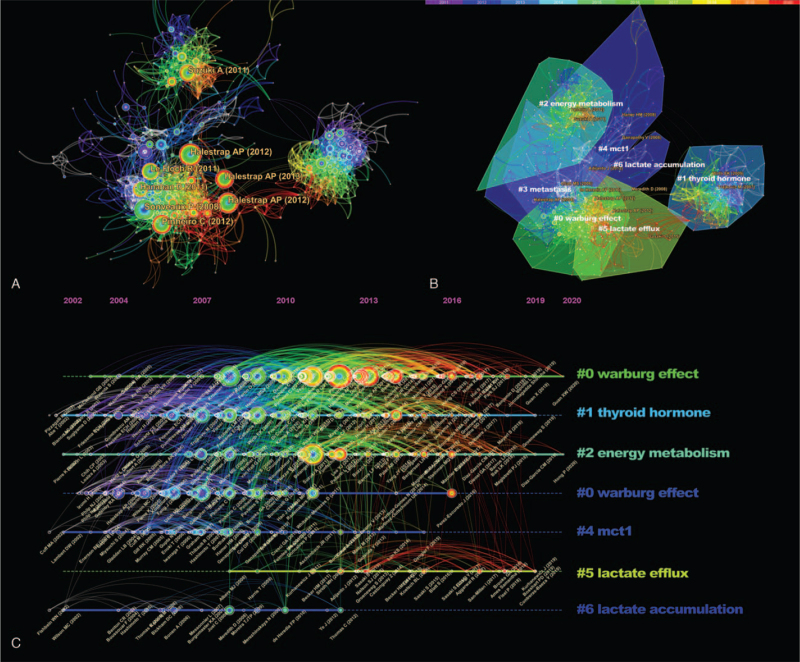
A. The network of co-citation references. B. Cluster analysis of cited references. (The size of the nodes is proportional to the citation frequency of the publication. The red ring in the node indicates when the maximum citation occurred, which is the hot spot. The nodes marked with purple outer rings are the turning points of the network. The color of the cluster boundary corresponds to the average age of the cluster. The warm color indicates that the cluster is newer, and the cold color indicates that the cluster is older.) C. Visualization analysis of clusters of co-citation references. (Timeline view).

When we write a paper, we usually cite previous research results and list them in the form of references. Co-citation of different references indicates the law of scientific development. A citation reference network with 653 nodes and 3720 links was constructed in Fig. 6A. “The monocarboxylate transporter family—Role and regulation,” published by Halestrap AP is the most cited publication with a frequency of 71 and centrality of 0.13, respectively.^[14]^ This article reviews in detail the physiological functions and regulation of different MCT family members, and prospects the role of MCT inhibitors in metabolism and their advantages as potential therapeutic agents (Supplemental_Dataset_S7 is CiteSpace data about distribution of co-cited references).

A large number of similar references can be categorized into several knowledge units by clustering analysis that can objectively reflect the main contents of each knowledge unit (Fig. 6B). Detailed information of 7 resultant clusters, including the size (the number of cited references), the silhouette value, the average publication year and the tags extracted from titles and abstracts by clustering labeling technique, were listed in Table 3. The silhouette values of all the clusters were >0.7, showing that the clustering results were convincing. Co-cited network based on 2010 to 2020 data was divided into seven sub-clusters (Fig. 6B), which were: #0 warburg effect, #1 thyroid hormone, #2 energy metabolism, #3 metastasis, #4 mct1, #5 lactate efflux, #6 lactate accumulation.

**Table 3:** Summary of cluster information.

Cluster ID	Size	Silhouette	Year	Tag (LLR)
#0	177	0.844	2012	Warburg effect; glycolysis
#1	136	0.981	2010	Thyroid hormone; mct8
#2	103	0.977	2011	Energy metabolism; astrocyte
#3	69	0.913	2007	Metastasis; tumor stroma
#4	59	0.937	2007	mct1; absorption
#5	40	0.954	2014	Lactate efflux; aerobic glycolysis
#6	18	0.970	2006	Lactate accumulation; horse

In order to understand the development path of MCTs research, we constructed a co-citation knowledge map of references (Fig. 6C). The horizontal coordinate of the timeline view is time, and the vertical coordinate is cluster ID. The time span and key articles of each cluster can be determined by placing the referenced articles in a cluster on the same horizontal line as depicted in Fig. 6C.

## 4 Discussion

### 4.1 Analysis of research development trend

Based on 2010 to 2020 data from WoSCC, a total of 1526 papers, from 354 institutions in 63 countries/regions, were published in 609 peer reviewed journals with 653 co-cited references. The scientiometric study included the analysis of the research status, development trend, and hotspots of the MCTs-related publications. The results of high-frequency keywords analysis show that the research of MCTs covers the aspects of the physiological roles of the different MCT isoforms, protein expression, glycolysis, poor prognosis, energy metabolism, apoptosis, tumor microenvironment (TME), energy metabolism reprogramming, and protein inhibitors, etc. Burst keywords from 2017 to 2020 were obtained as follows: tumor cell, breast cancer, apoptosis, cancer associated fibroblast (CAF), down regulation, cell proliferation, and AZD3659, which represent the development trend of MCTs. MCTs are widely expressed in different tumor types, not only in cancer cells but also in stromal cells. They exert multiple activities in cancer, including metabolic exchanges, metabolic signaling, and cancer metastasis. In a comprehensive study of 17 breast cancer cell lines, MCT4 was found to play an important role in the survival of breast cancer cells.^[33]^ Nowadays, TME and energy metabolism reprogramming have already been accepted as markers of cancer. Fiaschi et al^[34]^ reported the proof of metabolic reprogramming in CAF and human prostate cancer cells. Activation of CAF directed the metabolism towards glycolysis, the product of which was then transported back to cancer cells for tricarboxylic acid cycle and protein synthesis to promote cancer cells proliferation. It has been reported that the accumulation of lactate within tumors is associated with a poor clinical outcome.^[35,36]^ Izumi et al^[38]^ investigated the relationship between MCTs expression level and invasiveness of tumor cells and proved that the invasive rate of 11 types of lung cancer cell lines was significantly correlated with the expression levels of MCT1 and MCT4. In humans, high MCT1 and high MCT4 expression usually result in poor prognosis, whereas (aka SLC16A7) Isoform 2 in the MCT superfamily (MCT2) expression correlates with a favorable outcome.^[37]^ Metabolic adaptation of cancer cells is necessary for their growth and survival, suggesting that MCTs represent a novel opportunity of cancer treatment and are promising targets for the prevention of cancer invasion and metastasis.^[38–40]^

In addition, more and more researches on MCT2 and MCT8 emerged. MCT2 is located in neurons in the adult mice brains and is related to the postsynaptic density protein PSD-95. In the rat retina, MCT2 is heavily expressed throughout the inner retina, including the inner plexiform layer.^[41,42]^ At present, the pyruvate transport activity of MCT2 and its mechanism have been deeply studied. As a possible therapeutic target, it plays an important role in improving glaucoma and protecting eyesight.^[43,44]^ MCT8 is a thyroid hormone transporter with high affinity for T4 and T3.^[21]^ The mutation of MCT8 is the reason for severe X-linked psychomotor retardation and cause a severe neurodevelopmental disorder.^[45]^ Research about the mechanism of MCT8 and MCT2 deficiency, improving treatment strategies, and screening target drugs is another meaningful development trend.^[46,47]^

### 4.2 Analysis of research hotspots and frontiers

According to previous discussed burst keywords and clustering knowledge structures, MCTs is undoubtedly meaningful and necessary for the cancer research in the following aspects:

(1) Regulator of TME and metabolism. Metabolism plays an important role in maintaining intracellular homeostasis and responding to intracellular and extracellular stimuli. In order to maintain unlimited proliferation, tumor cells must adjust their metabolism and intake of nutrients, which is one of their characteristics. Because of the “Warburg effect,” cancer cells ferment glucose to lactate in the presence of oxygen. Aerobic glycolysis of tumor cells not only enables them intrinsic growth advantages, but also has external effects that disable immune surveillance. Targeting of glycolytic process may inhibit the pro-inflammatory response mediated by pro-inflammatory cytokines IL-17, IL-6, and IL-23, thus limiting tumorigenesis.^[48]^ However, it is noteworthy that if glycolytic pathway supporting function of T cells is targeted, blunt of the immune response may occur.^[49]^ In the premise of maintaining effective anti-tumor immunity, navigating metabolic pathways for anti-tumor therapy will be an important direction in future.^[50]^ Lactate transport into and out of cells via MCTs is crucial to maintain intracellular pH homeostasis, glycolysis, and extracellular acidic TME.^[51]^ And thus, regulation of glucose metabolism or lactate production and secretion by MCTs is a promising anti-cancer strategy.^[52,53]^
(2) Prognostic marker of cancer. Lactate shuttle across membranes is a common phenomenon in various types of tumor, including colon cancer, glioblastoma, breast cancer, prostate cancer, pancreatic cancer, and clear cell renal cell carcinoma, in which abnormal expression of MCT1, MCT2, and MCT4 have been confirmed.^[54,55]^ Since MCT1, MCT2, and MCT4 have important effects on the metabolism of lactate in tumor cells, they have been used as diagnostic biomarkers or prognostic factors for cancer prognosis and survival.^[56–58]^ MCT1 was proposed as a prognostic marker in non-small cell lung cancer and high expression of MCT1 in stromal cells correlated significantly with a poor disease-specific survival.^[59]^ MCT4 expression was found to be significantly associated with reduced overall survival in a cohort of 135 individuals with malignant pleural mesothelioma.^[60]^
(3) MCTs inhibitors. Table 4 summarizes several compounds that non-specifically inhibit MCTs. Phloretin, Quercetin, 4,4′-diisothiocyano-2,2′-stilbenedisulphonate, and α-cyano-4-hydroxycinnamate as well as its analogues are inhibitors that have been reported earlier and often miss targets due to their high affinity for other proteins.^[7,13,61–65]^ AR-C155858 is a dual MCT1/2 inhibitor that inhibits MCT2 when it is bound to ancillary protein basigin but not when it is bound to its preferred chaperone protein embigin.^[66–69]^ AZD3965, derived from AR-C155858, is an orally bioavailable specific inhibitor of MCT1. It has been evaluated as an antitumor agent in patients with prostate cancer, stomach cancer, and diffuse large b-cell lymphoma in a Phase I clinical trials.^[70–74]^ Since hypoxia-induced MCT4 is the major isoform in most highly aggressive tumor, scientists have been searching for MCT4-specific inhibitors. AstraZeneca developed a MCT4-specific inhibitor AZ93, but no pre-clinical data were released yet.^[75]^ Kobayashi et al^[76]^ identified a highly selective inhibitor of MCT4 that worked in a noncompetitive manner and was at least 15 times more selective for MCT4 than for MCT1. In a comprehensive study of 17 breast cancer cell lines, MCT4 was found to play an important role in the survival of breast cancer cells. Metabolic adaptation of cancer cells is necessary for their growth and survival, suggesting that MCT4 represents a novel opportunity of cancer treatment. There is no question that the exploitation of MCTs inhibitors as anti-cancer drugs to treat invasive cancers will be the frontier and possible trend of research in this field in the future.

**Table 4:** Summary of MCT inhibitors.

Inhibitors	MCT1 K_i_, μM	MCT2 K_i_, μM	MCT4 K_i_, μM	Other targets	References
Phloretin	5	14	41	Glucose transporters	^[13,61,62]^
Quercetin	10	5	40	ERβ	^[37,61,63]^
DIDS	434	No determined	No inhibition	Bicarbonate transporters	^[13,61,64]^
Simvastatin	>200	No determined	>200	HMG-CoA reductase	^[37,65]^
p-CMBS	25	Ni	25	Anion transporters	^[65,66]^
Lonidamine	36	36	40	Mitochondrial hexokinase	^[77,78]^
CHC	166	24	991	Mitochondrial pyruvate carrier	^[7,13,61,65]^
AR-C155858	0.002	<0.01	No inhibition	–	^[66–69]^
AZD3965	0.002	0.02	No inhibition	–	^[70–74]^
BAY-8002	0.085	∼0.425	No inhibition	–	^[79]^
Bindarit	No inhibition	No inhibition	30.2	Unknown	^[65]^
7ACC2	0.0011	No determined	No inhibition	Unknown	^[80,81]^
Syrosingopine	∼0.04	No determined	∼2.5	Unknown	^[82]^

## 5 Conclusions

In this paper, we performed a bibliometric analysis of 1526 MCTs related publications from 2010 to 2020 retrieved from WoSCC and visualization maps were drawn. The analysis network covers several aspects, including collaboration among various authors, areas, and institutions as well as contribution of journals. As shown in the results, research in this field is much deeper in USA and Europe, while the international collaboration is still limited. Meanwhile, collaboration between authors as well as institutions from different countries needs to be further strengthened in order to enhance the sharing of innovative research results and form a systematic theoretical framework. According to co-citation analysis, MCTs, as a drug target, is a research hotspot and targeting MCTs protein is still the focus of anti-cancer research.

However, there are also other challenges such as intricate metabolic interactions in the TME, lack of research to evaluate the clinicopathological significance of MCTs. Clinical exploitation of MCT inhibitors and the mechanism of lactic acid as signal molecule inducing angiogenesis are still important issues that need to be resolved.

## Author contributions

**Conceptualization:** Feifei Li, Yanli Zhang, Shuqi Wang.

**Data curation:** Youlong Yao, Yanli Zhang.

**Formal analysis:** Feifei Li, Ning Wang, Shuqi Wang.

**Methodology:** Yanli Zhang, Xueming Sun, Xiaoyan Wang.

**Software:** Youlong Yao, Yanli Zhang.

**Visualization:** Feifei Li, Yanli Zhang, Yulin You.

**Writing – original draft:** Yanli Zhang, Ning Wang, Shuqi Wang.

**Writing – review & editing:** Feifei Li, Xueming Sun, Xiaoyan Wang.

## Supplementary Material

Supplemental Digital Content

## Supplementary Material

Supplemental Digital Content

## Supplementary Material

Supplemental Digital Content

## Supplementary Material

Supplemental Digital Content

## Supplementary Material

Supplemental Digital Content

## Supplementary Material

Supplemental Digital Content

## Supplementary Material

Supplemental Digital Content
